# Efficient Generation of Rat Induced Pluripotent Stem Cells Using a Non-Viral Inducible Vector

**DOI:** 10.1371/journal.pone.0055170

**Published:** 2013-01-31

**Authors:** Claudia Merkl, Anja Saalfrank, Nathalie Riesen, Ralf Kühn, Anna Pertek, Stefan Eser, Markus Sebastian Hardt, Alexander Kind, Dieter Saur, Wolfgang Wurst, Antonio Iglesias, Angelika Schnieke

**Affiliations:** 1 Chair of Livestock Biotechnology, Technische Universität München, Freising, Germany; 2 Klinikum Rechts der Isar II, Technische Universität München, Munich, Germany; 3 Institute for Developmental Genetics, Helmholtz Center Munich, Munich, Germany; 4 Technische Universität München, Munich, Germany; 5 Deutsches Zentrum für neurodegenerative Erkrankungen e.V., Munich, Germany; 6 Small Molecule Research - Discovery Technologies, Pharma Research and Early Development, F. Hoffmann-La Roche Ltd., Basle, Switzerland; Indian Institute of Toxicology Reserach, India

## Abstract

Current methods of generating rat induced pluripotent stem cells are based on viral transduction of pluripotency inducing genes (*Oct4*, *Sox2*, c-*myc* and *Klf4*) into somatic cells. These activate endogenous pluripotency genes and reprogram the identity of the cell to an undifferentiated state. Epigenetic silencing of exogenous genes has to occur to allow normal iPS cell differentiation. To gain more control over the expression of exogenous reprogramming factors, we used a novel doxycycline-inducible plasmid vector encoding Oct4, Sox2, c-Myc and Klf4. To ensure efficient and controlled generation of iPS cells by plasmid transfection we equipped the reprogramming vector with a bacteriophage φC31 attB site and used a φC31 integrase expression vector to enhance vector integration. A series of doxycycline-independent rat iPS cell lines were established. These were characterized by immunocytochemical detection of Oct4, SSEA1 and SSEA4, alkaline phosphatase staining, methylation analysis of the endogenous Oct4 promoter and RT-PCR analysis of endogenous rat pluripotency genes. We also determined the number of vector integrations and the extent to which reprogramming factor gene expression was controlled. Protocols were developed to generate embryoid bodies and rat iPS cells demonstrated as pluripotent by generating derivatives of all three embryonic germ layers *in vitro*, and teratoma formation *in vivo*. All data suggest that our rat iPS cells, generated by plasmid based reprogramming, are similar to rat ES cells. Methods of DNA transfection, protein transduction and feeder-free monolayer culture of rat iPS cells were established to enable future applications.

## Introduction

The rat has long been an invaluable animal model in many biomedical research fields, including behavioral studies, cardiovascular disease, immunology, transplantation, toxicology and pharmacology [1], [2], [3], [4]. However the use of rats has been hindered by the lack of embryonic stem (ES) cells and the consequent difficulty in generating animals with precise genetic modifications. Derivation of germline competent rat ES cells [5], [6] and their functional equivalent, induced pluripotent stem cells [7], [8] thus represents a major step forward.

Induced pluripotent stem (iPS) cells can be derived from somatic cells by forced expression of exogenous transcription factors, notably Oct4, Sox2, c-Myc and Klf4 [9]. These activate endogenous pluripotency genes and reprogram the cell to a pluripotent state. ES and iPS cells have now enabled the generation of rats with gene targeted inactivation of p53, protease-activated receptor-2 and hypoxanthine phosphoribosyltransferase [10], [11], [12], [13]. Directed differentiation of rat pluripotent stem cells also provides a source of cell types such as cardiomyocytes [14], which are useful for toxicological screening, research into tissue regeneration and development of organ repair procedures.

Rat iPS cell lines have been derived from different rat strains and a variety of somatic cells, including embryonic fibroblasts, neural precursor cells, bone marrow cells, liver progenitor cells, and ear fibroblasts [8], [7], [15], [16]. However this work has been based on retroviral or lentiviral vectors that have drawbacks. Establishment of a true self-sustaining pluripotent state, independent of exogenous reprogramming factor expression, requires epigenetic silencing of the exogenous genes. Virally transduced genes are frequently silenced in the host cell, but persistence or reactivation of factor expression interferes with differentiation, and c-Myc expression has led to tumor formation in iPS derived offspring in mice [17]. Next generation iPS cells must therefore incorporate tight control of transgene expression. The limited capacity of retroviral vectors also means that individual retroviruses are commonly used to introduce reprogramming factors. Multiple independent infections of each cell are thus required to deliver the full complement of factors and many cells may not receive an ideal, equimolar ratio of each gene [18].

Here we report iPS cell generation using a non-viral vector containing the murine reprogramming factors Oct4, Sox2, c-Myc and Klf4 controlled by a bidirectional doxycycline-inducible promoter.

## Materials and Methods

Animal experiments were approved by the Government of Upper Bavaria and performed according to the German Animal Welfare Act and European Union Normative for Care and Use of Experimental Animals (permit number: Az. 209.1/211-2531-114-03). Chemicals were obtained from Sigma Aldrich, and cell culture media and supplements from PAA Laboratories or Gibco Life Technologies unless otherwise specified. Oligonucleotide sequences are shown in Tables S1, S2, S3.

### Plasmids

The reprogramming cassette of pReproII-attB was assembled by standard cloning and PCR methods using the P_bi-1_ promoter from pBI-5 (Clontech) and cDNAs for murine Oct4, Sox2, Klf4 and c-Myc (ImaGenes). Two coding regions, linked by a T2A peptide sequence [19], [20], were placed on either side of the P_bi-1_ promoter. Each bicistronic coding region (Oct4-T2A-c-Myc or Sox2-T2A-Klf4) is preceded by a hybrid intron [21] and terminated by the bovine growth hormone (bGH) polyadenylation signal. The reprogramming unit was combined with a CAG (cytomegalovirus early enhancer element and chicken beta-actin promoter) promoter driven expression cassette for rtTA2(S)-M2 and tTS^KRAB^ from plasmid pRTS-1 [22], in which the Eμ enhancer was replaced by the cytomegalovirus early enhancer. A synthetic DNA element containing a 53 bp attB site, which can be recognized by φC31 integrase, was added to generate pReproII-attB. The φC31 integrase expression plasmid pCAG-C31Int(NLS) has been described previously [23].

### Generation of Rat iPS Cells

**Figure 1:**
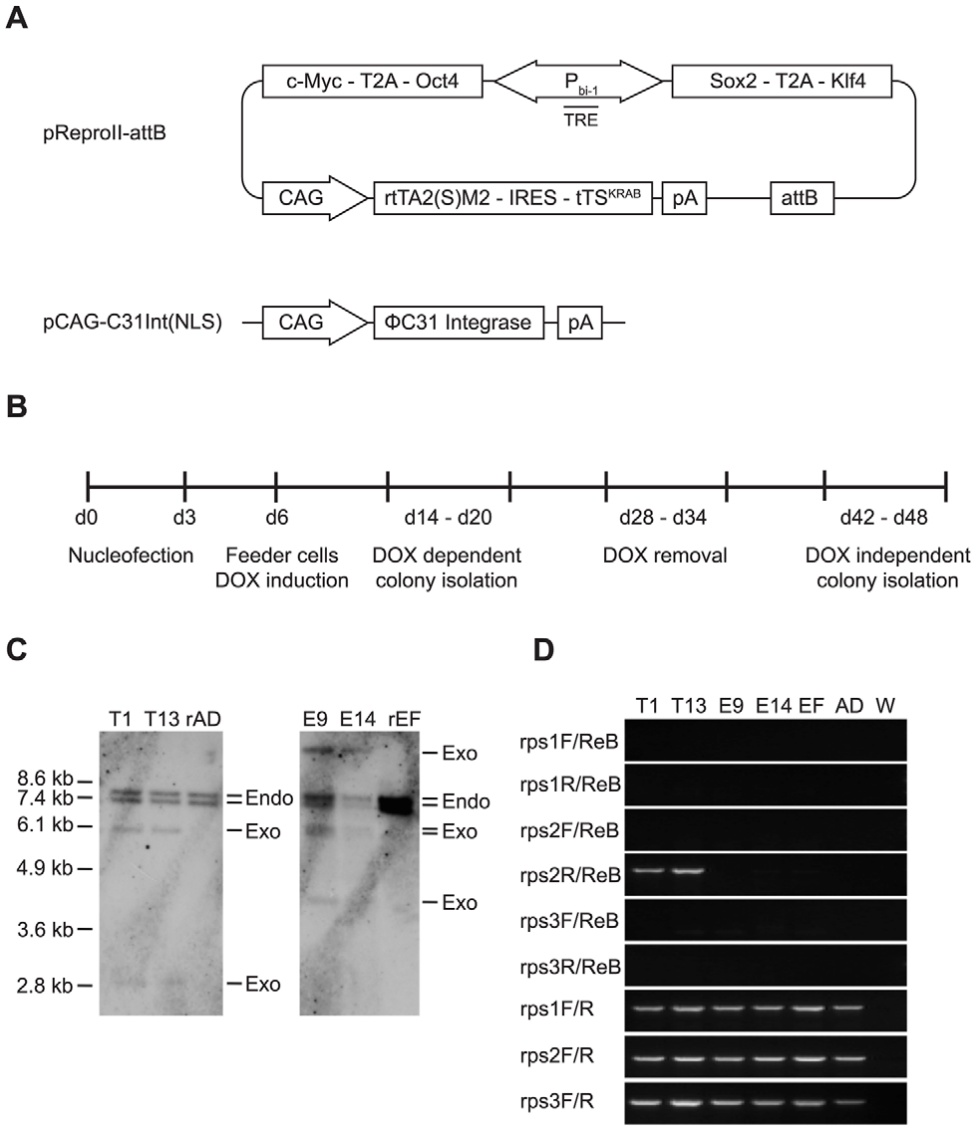
Generation and analysis of iPS cells. (A) Schematic representation of the reprogramming vector pReproII-attB and the φC31 integrase expression plasmid pCAG-C31Int(NLS). Indicated are the four transcription factors Oct4, Sox2, c-Myc and Klf4 linked by T2A peptides, the bidirectional doxycycline-inducible promoter P_bi-1_, consisting of two copies of the minimal CMV promoter linked by a tet-responsive element (TRE), the expression cassette for the reverse tetracycline controlled transactivator rtTA2(S)-M2 and the tet repressor-KRAB fusion protein tTS^KRAB^ linked by an internal ribosome entry site (IRES), the chicken beta-actin enhancer/promoter (CAG) and the attB site. The plasmid pCAG-C31Int(NLS) contains a constitutive expression cassette for the φC31 integrase under the control of the CAG promoter. (B) Time line of rat iPS cell generation. DOX: doxycycline, d: day. (C) Southern blot analysis of four doxycycline-dependent rat iPS cell lines. A Klf4 probe was used that detects two endogenous fragments (Endo) in source cells rADMSC (rAD) and rEF as well as exogenous Klf4 (Exo) in iPS cell lines. (D) PCR analysis of rat pseudo-attP sites in lines T1, T13, E9 and E14. Primers flanking known rat pseudo-attP sites (rps1F/R, rps2F/R, rps3F/R) were combined with a primer binding in pReproII-attB (ReB). Rat EF, ADMSC (AD) and water (W) were used as negative controls.

Adipose tissue-derived mesenchymal stem cells (rADMSC) were isolated from subcutaneous fat, and fibroblasts from ear tissue (rEFs) of Fischer and Wistar rats according to standard methods. The passage number of the source cells was between P2 and P3, both showed typical fibroblast-like morphology. For the generation of iPS cells, 5×10^5^ rADMSCs were nucleofected with 1 µg reprogramming vector pReproII-attB and 1 µg φC31 integrase expression vector pCAG-C31Int(NLS) (see Figure 1A) using the Nucleofector II device (Lonza) with program U-023 and the Human MSC Nucleofector Kit (Lonza). 5×10^5^ rEFs were nucleofected with 3 µg pReproII-attB and 3 µg pCAG-C31Int(NLS) using program A-024 and the Basic Nucleofector Kit for Primary Mammalian Fibroblasts (Lonza). Cells were plated onto tissue culture flasks on day 0 and nucleofection repeated on day 3. Transfected cells were transferred onto mitomycin C inactivated mouse embryonic fibroblasts (MEFs) on day 6. A schematic overview is shown in Figure 1B. Rat iPS cells were derived in two different media. N2B27-3i medium: 1∶1 mixture of N2 medium (DMEM/F12, 1×N2 supplement, 100 µg/ml BSA fraction V) and B27 medium (Neurobasal medium, 1×B27 supplement w/o retinoic acid, 2 mM Glutamax) supplemented with 0.1 mM 2-mercaptoethanol, 20% Knockout Serum Replacement, 1000 U/ml hLIF (produced in house), 3 µM GSK3β inhibitor CHIR99021 (Axon Medchem), 0.5 µM MEK1/2 inhibitor PD0325901 (Axon Medchem), 0.5 µM ALK5 inhibitor A83-01 (Biotrend). N2B27-2i medium: 1∶1 mixture of N2 medium and B27 medium (see above) supplemented with 0.1 mM 2-mercaptoethanol, 1000 U/ml hLIF, 3 µM GSK3β inhibitor CHIR99021, 0.5 µM MEK1/2 inhibitor PD0325901. Doxycycline (1.5 µg/ml) was added to culture media to induce expression of reprogramming factors.

### General Cell Culture

Rat adipose tissue-derived mesenchymal stem cells (rADMSC) were cultured in MSC medium (MEM α, 10% FCS). Rat ear fibroblasts (rEF) were cultured in EF medium (DMEM, 15% FCS, 1× non-essential amino acids, 1× sodium pyruvate, 2 mM Glutamax, 5 ng/ml bFGF (Promokine)). Mouse embryonic fibroblasts (MEF) were cultured in DMEM+ medium (DMEM, 10% FCS, 1× non-essential amino acids, 2 mM Glutamax, 1× sodium pyruvate). Rat iPS cells were maintained on mitomycin C inactivated MEFs in N2B27-2i or N2B27-3i medium. iPS cells were routinely subcultured every 3 to 4 days by flushing loosely attached colonies off the feeder layer and dissociation by Accutase in suspension. For feeder-free monolayer culture, plates were coated with either 0.1% gelatin from bovine skin in PBS, 5 µg/ml fibronectin in PBS, 200 µg/ml rat tail collagen type I (Serva) in H_2_O, 2% growth factor reduced Matrigel (BD Biosciences) in DMEM/F12, 2% Geltrex (Invitrogen) in DMEM/F12 or 4.2 µg/ml laminin (Roche) in PBS for 2 h at 37°C.

### Isolation of Genomic DNA and RNA

Genomic DNA for bisulfite sequencing and PCR was isolated using the GenElute Mammalian Genomic DNA Miniprep Kit (Sigma). DNA for Southern blot analysis was obtained by standard phenol/chloroform extraction. RNA was isolated using either Trizol (Invitrogen), or the High Pure RNA Isolation Kit (Roche), and genomic DNA removed by treatment with the Turbo DNA-free Kit (Ambion) according to the manufacturer’s instructions.

### RT-PCR

RNA was reverse transcribed with random hexamer oligonucleotides using SuperScript III (Invitrogen) according to the manufacturer’s protocol. PCR with GoTaq DNA polymerase (Promega) was performed using oligonucleotides listed in Table S1. Thermal cycling conditions were: 94°C, 2 min; 30 cycles of 94°C for 30 s, 58°C for 30 s, 72°C for 1 min; then final elongation 72°C for 5 min. Quantitative PCR was performed using a 7500 Fast Real-Time PCR System and the SYBR Green PCR Master Mix (Applied Biosystems) with oligonucleotides listed in Table S1, according to the manufacturer’s instructions. Thermal cycling conditions were: 95°C, 10 min; 40 cycles of 95°C for 15 s, 60°C for 1 min. Expression of the exogenous reprogramming factors was calculated with the ΔΔCT method and expressed as fold change relative to the corresponding cell line with doxycycline induction.

### Southern Blot Analysis

10 µg genomic DNA was digested with *BglII*, separated by gel electrophoresis and transferred to Hybond-N+ membrane by capillary blotting. The hybridization probe was generated by PCR with GoTaq DNA polymerase (Promega) incorporating alkali-labile digoxigenin-11-dUTP (Roche). The 1308 bp Klf4 probe was amplified from pReproII-attB with primers prKlf4_F and prKlf4_R (see Table S3). Thermal cycling conditions were: 94°C, 2 min; 35 cycles of 94°C for 30 s, 58°C for 30 s, 72°C for 90 s; then final elongation 72°C for 5 min. Hybridization using DIG Easy Hyb (Roche) and probe detection using anti-digoxigenin antibody Fab fragments conjugated with alkaline phosphatase (Roche) were performed according to the manufacturer’s instructions.

### PCR for Pseudo-attP Sites

Pseudo-attP site PCR analysis was performed as described [24] using published oligonucleotides for rat pseudo-attP sites rps1, rps2 and rps3 (see Table S2). Each PCR assay used one primer within the reprogramming vector and one primer in the genomic DNA sequence upstream or downstream of rps1, 2 or 3 sites. PCR was performed with GoTaq DNA polymerase (Promega) according to the manufacturer’s instructions. Thermal cycling conditions were: 94°C, 2 min; 35 cycles of 94°C for 30 s, 58°C for 30 s, 72°C for 2 min; then final elongation 72°C for 5 min. PCR products from rat iPS cell lines T1 and T13 were subcloned into pJet1.2/blunt (Fermentas) and the DNA sequence determined.

### Alkaline Phosphatase and Immunocytochemistry

Cells were fixed with 4% paraformaldehyde. Alkaline phosphatase staining was performed with SIGMA*FAST* BCIP/NBT according to the manufacturer’s instructions. Immunocytochemistry of undifferentiated and differentiated iPS cells was performed with primary antibodies against Oct4 (1∶100; sc-8628, Santa Cruz), SSEA1 (1∶200; sc-21702, Santa Cruz), SSEA4 (1∶100; sc-21704, Santa Cruz), albumin (1∶100; A0001, Dako), sarcomeric α-actinin (1∶250; EA-53, Sigma) or βIII-tubulin (1∶250; SDL.3D10, Sigma) followed by goat anti-mouse IgM-FITC (1∶200; sc-2082, Santa Cruz), chicken anti-goat IgG-FITC (1∶200; sc-2988, Santa cruz), goat anti-mouse IgG-FITC (1∶200; sc-2010, Santa Cruz), Alexa Fluor 594 goat anti-rabbit IgG (1∶750; A11012, Invitrogen) secondary antibodies. Nuclei were stained with Hoechst 33258.

### Bisulfite Sequencing

500 ng genomic DNA was treated with the Epitect Bisulfite Kit (Qiagen) or Epimark Bisulfite Conversion Kit (NEB) according to the manufacturer’s instructions. A 206 bp region of the endogenous rat Oct4 promoter (−1495 to −1290) was amplified by PCR from bisulfite converted genomic DNA using primers BS-Oct4_F and BS-Oct4_R [8] (see Table S3). PCR was performed with GoTaq DNA polymerase (Promega). Thermal cycling conditions were: 94°C, 2 min; 35 cycles of 94°C for 30 s, 55°C for 30 s, 72°C for 1 min; then final elongation 72°C for 5 min. PCR fragments were subcloned into the vector pJet1.2/blunt (Fermentas) and the DNA sequence of five individual clones determined. Bisulfite sequencing data were analyzed with the online tool QUMA [25].

### Karyotype Analysis

Rat iPS cells in log phase were treated with 10 µg/ml colcemid for 4 h. Cells were collected, treated with Accutase to obtain a single cell suspension, incubated for 12 min at room temperature in 75 mM KCl and fixed with ice cold methanol/acetic acid (3∶1). Metaphase preparation and chromosome counting was performed by CHROM*Bios* GmbH (Nussdorf, Germany).

### Embryoid Body (EB) Formation

Embryoid bodies were generated either by growth in suspension, or “colony EB” culture. For suspension culture, iPS cells were dissociated with Accutase, resuspended at 4×10^6^ cells per 15 ml EB medium I (50% N2B27-2i, 50% DMEM+) and cultured in 10 cm non-adhesive culture dishes. For colony EB culture, loosely attached iPS colonies were flushed off the feeder layer and transferred into 10 cm non-adhesive culture dishes in EB medium I. For both methods, the medium was changed to EB medium II (30% N2B27-2i, 70% DMEM+) after 48 h. A further 48 h later, medium was changed to DMEM+ and EBs cultured for an additional 4 days in non-adhesive culture dishes. After 8 days EBs were analyzed or allowed to attach to gelatin-coated tissue culture plates in DMEM+ medium.

### Teratoma Formation

4–5×10^6^ rat iPS cells from line T1/64 were resuspended in N2B27-2i, mixed with high density Matrigel (BD Bioscience) and injected subcutaneously into NOD scid gamma (NSG) mice. Teratomas were harvested after 25 days, fixed in 4% paraformaldehyde, embedded in paraffin and sectioned. Sections were stained with hematoxylin and eosin (H&E) according to standard protocols.

### Transfection of Rat iPS Cells

Rat iPS cells were transfected with Nanofectin (PAA), or Lipofectamine 2000 (Invitrogen) as monolayer cultures on 2% Geltrex (Invitrogen) in 12 well plates according to the manufacturer’s instructions using the GFP expression plasmid pmaxGFP (Lonza). Nucleofection was performed using the Nucleofector II device (Lonza) and the Mouse Embryonic Stem Cell Kit (Lonza) with program A-024 according to the manufacturer’s instructions.

### Production of Recombinant NLS-Cherry-9R Protein and Protein Transduction

The expression vector pTriEx-Cherry encodes the red fluorescent protein NLS-Cherry-9R. NLS-Cherry-9R contains a 6xHis tag, the SV40 Large-T nuclear localization signal (NLS) at the N-terminus and a protein transduction domain consisting of 9 arginine residues (9R) at the C-terminus of the mCherry red fluorescent protein. The pTriEx-Cherry expression cassette was assembled by standard PCR methods. Recognition sites for the restriction enzyme *BspHI* and *XhoI*, the 6xHis tag, NLS sequence and 9R were added to the mCherry fluorescent reporter gene (Clontech) coding region with long oligonucleotides using Phusion Polymerase (Finnzymes). The mCherry translational start codon was mutated from ATG to GTG, which also encodes the C-terminal valine of the NLS to avoid translation of untagged protein. The cassette was inserted between the *NcoI* and *XhoI* restriction sites of pTriEx-HTNC (Addgene plasmid 13763, [26]) to generate pTriEx-Cherry. Expression in bacteria and purification of NLS-Cherry-9R was performed according to [26]. Protein transduction was performed with iPS cells on MEF feeder cells, in suspension culture in 15 ml Falcon tubes, or in monolayer culture on 2% Geltrex using 5 µM recombinant protein for 4 or 24 h.

## Results

### Generation of Doxycycline-dependent Rat iPS Cells

Two cell types from two rat strains were used to generate iPS cells: adipose tissue-derived mesenchymal stem cells (rADMSC) and ear fibroblasts (rEF) from Fischer and Wistar rats. Cells were cotransfected with the reprogramming vector pReproII-attB and the φC31 integrase expression vector pCAG-C31Int(NLS) (see Figure 1A). pReproII-attB contains the minimal bidirectional doxycycline-inducible promoter P_bi-1_ [27], which directs expression of the murine reprogramming factors Oct4, c-Myc, Klf4 and Sox2 as bicistronic mRNAs. The pReproII-attB vector also encodes other necessary components of the Tet-On system: the tetracycline-controlled transactivator rtTA2(S)-M2 and the tetracycline-regulated repressor tTS^KRAB^ under the control of the constitutive CAG promoter [28], [22], [29]. An attB site is also included to facilitate φC31 integrase-mediated integration at pseudo-attP sites in the host genome [30], [31].

We found that double nucleofection (on days 0 and 3) resulted in more efficient generation of rat iPS cells than a single nucleofection step. Six days after initial nucleofection, cells were transferred onto a feeder layer in N2B27-3i medium containing doxycycline to induce expression of the exogenous reprogramming factors. Colonies with rat ES/iPS cell-like morphology appeared 8 to 10 days after doxycycline induction. Individual colonies were manually picked between day 14 and 20, transferred to multiwell plates and expanded. The outline scheme is shown in Figure 1B. A total of 37 iPS cell lines were established from Fischer rat ADMSCs and EFs and 18 lines from Wistar rat ADMSCs and EFs. Results and efficiencies are summarized in Table 1. At this stage, growth of each line relied on continued presence of doxycycline and expression of exogenous reprogramming factors.

**Table 1:** Doxycyline-dependent rat iPS cell lines.

Rat strain	Cell type	Isolated colonies	Established lines	Efficiency
Fischer334	rADMSC	129	21	16.2%
Fischer334	rEF	73	16	21.9%
Wistar	rADMSC*	17	7	41.1%
Wistar	rEF	96	11	11.4%

* Established in N2B27/2i medium instead of N2B27/3i.

### Analysis of Vector Integration

We used a non-viral vector equipped with an attB phage φC31 integrase recognition site (see Figure 1A) to facilitate integration into the rat genome. Three preferred integration sites, so called pseudo-attP sites rps1, rps2 and rps3, have been identified in the rat genome [24].

Two Fischer iPS cell lines, T1 and T13, and two Wistar iPS cell lines, E9 and E14, were analyzed by Southern blot to determine the number of vector integrations in each. We identified two (T1, T13) or four (E9, E14) integrations as shown in Figure 1C. To determine whether these were at known pseudo-attP sites (rps) we performed PCR analysis similar to that previously described [24]. φC31-mediated integration can occur in either forward or reverse orientation, we therefore analyzed integration in both directions. None were found at sites rps1 or rps3. Screening of rps2 sites revealed integration in reverse orientation in lines T1 and T13. The wild-type fragment spanning rps1, 2 and 3 was amplified in all lines, indicating that the rps2 integration in lines T1 and T13 had occurred at one allele (see Figure 1D). Sequence analysis of the PCR fragment from lines T1 and T13 confirmed vector integration into the rps2 site on chromosome 1q41. The other integrations did not occur at the known three sites.

### Generation of Doxycycline-independent Rat iPS Cell Lines

**Figure 2:**
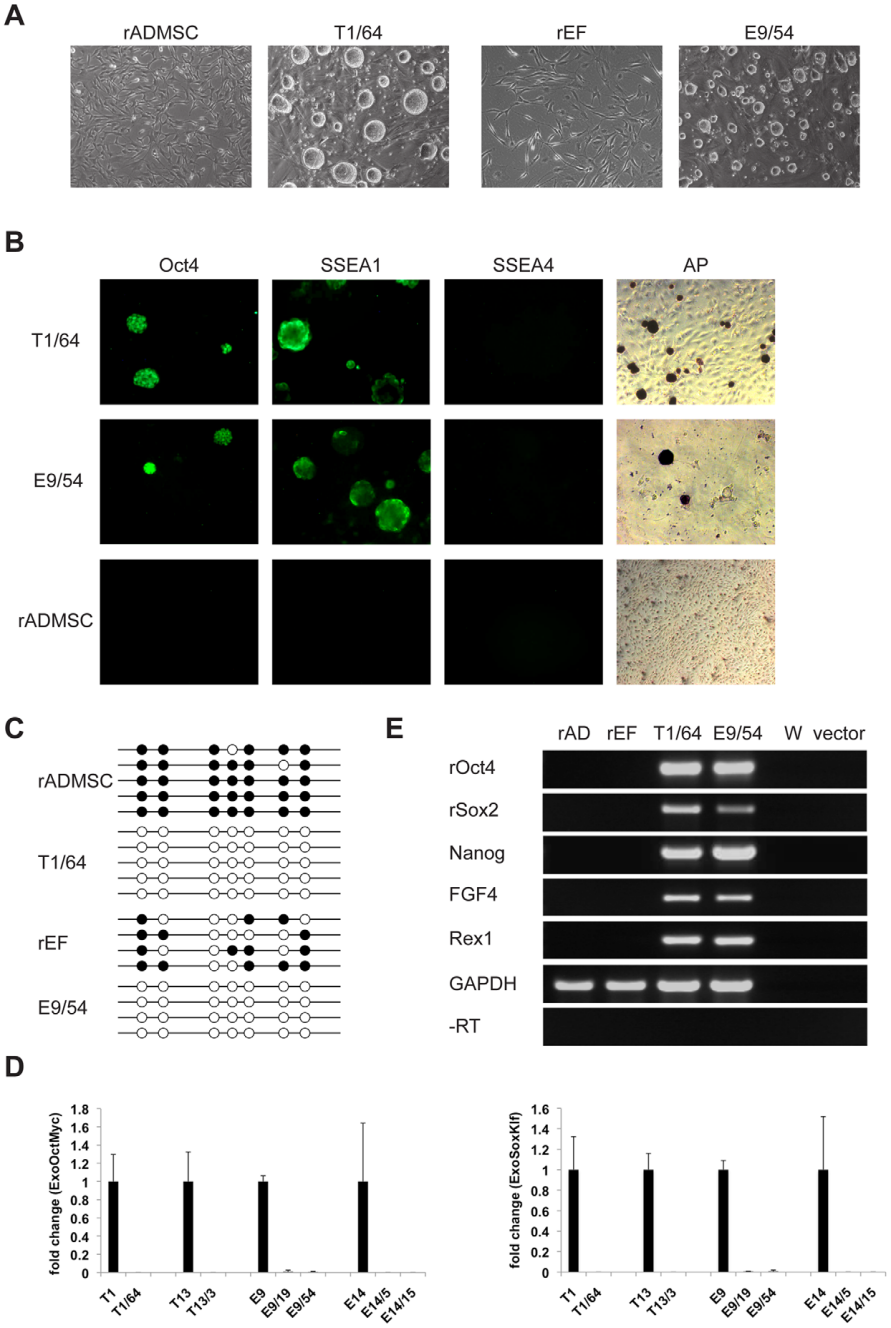
Analysis of doxycycline-independent rat iPS cell lines. (A) Morphology of somatic cell types used to generate rat iPS cells (rADMSC, rEF) and typical spheroidal morphology of doxycycline-independent iPS cell lines T1/64 and E9/54 on feeder cells (10× magnification). (B) Immunocytochemical and alkaline phosphatase staining of iPS lines T1/64 and E9/54 and rADMSC (10× and 5× magnification). (C) Methylation analysis of rat Oct4 promoter region (−1495 to −1290) in source cells rADMSC, rEF and iPS lines T1/64 and E9/54. Circles indicate CpG methylation sites, dark shading represents methylated and open shading non-methylated. (D) Quantitative RT-PCR analysis of exogenous reprogramming factor expression. Fold change in bicistronic mRNA detected in doxycycline-independent (T1/64, T13/3, E9/19, E9/54, E14/5, E14/15) iPS cells is shown relative to each parental doxycycline-dependent line (T1, T13, E9, E14) as indicated. Left: Oct4-T2A-c-Myc bicistronic fragment. Right: Sox2-T2A-Klf4 bicistronic fragment. Data was normalized to GAPDH expression. (E) Expression analysis of endogenous pluripotency factors Oct4, Sox2, Nanog, FGF4 and Rex1 in somatic source cells rADMSC (rAD) and rEF and doxycycline-independent iPS cell lines T1/64 and E9/54. GAPDH control, water (W), reprogramming vector and reverse transcriptase enzyme (-RT) negative controls are indicated.

Two doxycycline-dependent iPS lines from Fischer ADMSCs (T1, T13), and two from Wistar EF (E9, E14) were used for further experiments. Initially iPS cells were cultured in N2B27-3i medium with doxycycline for 14 days, then doxycycline induction withdrawn. 263 colonies were isolated between days 14 and 24 after doxycycline removal from lines T1 and T13 and expanded in N2B27-3i medium (see Figure 1B). 10 doxycycline-independent iPS cell lines were established. This rather low efficiency (<4%) led us to investigate alternative culture media. Twelve media compositions were tested, these differed in the presence or absence of Knockout Serum Replacement, inhibitor combinations, addition of Thiazovivin, ROCK inhibitor Y27632 or ascorbic acid (see Table S4). Best results were obtained with N2B27-2i medium, which differs from N2B27-3i in that it lacks ALK5 inhibitor A83-01 and Knockout Serum Replacement. Using N2B27-2i, 15 of 40 (37.5%) colonies from Fischer line T13 and 39 of 114 colonies from Wistar lines E9 and E14 (22.2% to 36.5%) were established as doxycycline-independent iPS cell lines, representing a considerable improvement over N2B27-3i. Detailed results and efficiencies are shown in Table 2. This, together with reduced spontaneous differentiation and a more stable ES-like cell morphology led us to use N2B27-2i medium for further culture. Examples of cell morphology before reprogramming and after doxycycline removal are shown in Figure 2A. Comparison of our reprogramming efficiencies with rat iPS cells generated by viral methods or by φC31 integrase-based reprogramming of murine and human cells is shown in Table 3.

**Table 2:** Doxycycline-independent rat iPS cell lines.

iPS line	Medium	Isolated colonies	Established lines	Efficiency
T1 (Fischer)	N2B27/3i	155	6	3.8
T13 (Fischer)	N2B27/3i	108	4	3.7
T13 (Fischer)	N2B27/2i	40	15	37.5
E9 (Wistar)	N2B27/2i	96	35	36.5
E14 (Wistar)	N2B27/2i	18	4	22.2

**Table 3:** Comparison of reprogramming efficiencies.

Publication	Method	Factors	Cell type	Starting cell number	Colonies	iPS lines	Reprogramming efficiency relative to starting cell number
Liskovykh et al. 2011	LV	mOSKM	REF	1.5×10^5^	48 isolated	4.6–13.9	0.003–0.009%
Liskovykh et al. 2011	LV	mOSKM	REF	1.5×10^5^	48 isolated	7.74–9.25	0.005–0.006%
Hamanaka et al., 2011	LV	mOSK	REF	Not stated	Not stated	8	Not stated
Chang et al., 2010	RV	OSKMN	rNPC	5×10^4^ cells	93 isolated, 51 AP+, 20% correct morphology (REF as feeder cells); 53 isolated, 36 AP+, 20% correct morphology (MEF as feeder cells)	Not stated	0.02% (AP+, correct morphology, on REF); 0.014% (AP+, correct morphology, on MEF)
Chang et al., 2010	RV	OSKM	REF	5×10^4^ cells	67 isolated, 43 AP+, 20% correct morphology (REF as feeder cells); 34 isolated, 20 AP+, 20% correct morphology (MEF as feeder cells)	Not stated	0.017% (AP+, correct morphology, on REF); 0.008% (AP+, correct morphology, on MEF)
Liao et al., 2009	RV	hOSKM	rEF	5×10^5^	27 colonies	Not stated	0.0054%
Liao et al., 2009	RV	hOSKM	rBMC	5×10^5^	12 colonies	Not stated	0.0024%
Li et al., 2009	RV	mOSK	rLPC	1×10^5^	Not stated	Not stated	0.4% (AP+ colonies)
Karow et al., 2011	C31	mOSKM	MEF	1×10^6^	Not stated	Not stated	0.01% (AP+ or SSEA1+)
Karow et al., 2011	C31	mOSKM	mASC	1×10^6^	Not stated	Not stated	0.014% (AP+ or SSEA1+)
Ye et al., 2010	C31	OSKM	MEF	1×10^6^	29 colonies	17 lines	0.0017%
This publication	C31	mOSKM	rEF	1×10^6^ cells (Fischer+Wistar)	169 colonies (+DOX)	27 lines	0.0027% (+DOX)
This publication	C31	mOSKM	rADMSC	1×10^6^ cells (Fischer+Wistar)	146 colonies (+DOX)	28 lines	0.0028% (+DOX)
This publication	C31	mOSKM	rEF	5×10^5^ cells (Wistar)	114 colonies (−DOX, 2i)	39 lines	0.0078% (−DOX, 2i)
This publication	C31	mOSKM	rADMSC	5×10^5^ cells (Fischer)	303 colonies (−DOX, 2i/3i)	25 lines	0.005% (−DOX, 2i/3i)

LV: Lentivirus; RV: Retrovirus; C31: ΦC31 Integrase; O: Oct4; S: Sox2; K: Klf4; M: c-Myc; N: Nanog; r: rat; m: mouse; h: human; REF: rat embryonic fibroblasts, MEF: mouse embryonic fibroblasts; EF: ear fibroblasts; ADMSC: adipose tissue derived mesenchymal stem cells; NPC: Neural precursor cells; LPC: liver precursor cells; BMC: bone marrow cells.

### Expression of Exogenous Reprogramming Factors in Doxycycline-dependent and -Independent Rat iPS Cell Lines

We compared the expression of exogenous reprogramming factors in the parental doxycycline-dependent rat iPS cell lines (T1, T13, E9 and E14) in the presence of doxycycline, with six doxycycline-independent subclones (T1/64, T13/3, E9/19, E9/54, E14/5 and E14/15). Quantitative RT-PCR specific for bicistronic Oct4-T2A-c-Myc or Sox2-T2A-Klf4 mRNAs showed high exogenous factor expression in doxycycline-dependent lines, and 100- to 1,000,000-fold less in doxycycline-independent subclones, see Figure 2D. This, together with an undifferentiated morphology, indicated successful activation of the endogenous pluripotency program.

### Pluripotency Markers, Methylation Status and Karyotype Analysis

We analyzed the expression of endogenous pluripotency marker genes in two doxycycline-independent iPS cell lines: Fischer iPS line T1/64 and Wistar iPS line E9/54. Oct4, Sox2, Nanog, FGF4 and Rex1 expression was analyzed by RT-PCR using rat-specific primers. As Figure 2E shows, both iPS lines expressed these key markers, while the source rADMSC and rEF cells did not. Both lines also expressed the stem cell marker alkaline phosphatase (AP) (see Figure 2B). Immunocytochemical analysis revealed that both lines T1/64 and E9/54 were Oct4,SSEA1 positive and SSEA4 negative (see Figure 2B), which accords with previous descriptions of rat ES and iPS cells [5], [8], [7]. Source cells were Oct4, SSEA1, SSEA4 and AP negative (see Figure 2B).

Another hallmark of successful reprogramming is the demethylation of promoter regions of key pluripotency genes such as Oct4 and Nanog [32], [33]. We performed bisulfite sequencing of a 206 bp region (−1495 to −1290) of the rat Oct4 promoter, which contains 7 CpG sites [8]. In rADMSCs 6 to 7 CpG sites were methylated, in rEFs 3 to 5 were methylated, whereas none were methylated in iPS cell lines T1/64 and E9/54 (see Figure 2C). This further confirmed reprogramming to a self-sustaining pluripotent state.

We then performed karyotype analysis. Rat iPS cell line T1/64 showed a normal karyotype (2n = 42) in 80% metaphase spreads examined. Line E9/54 was mostly polyploid. Diploid metaphase spreads were analyzed and 20% showed normal karyotype (2n = 42). These results accord with previous reports of rat ES and iPS cell karyotypic variability [5], [6], [10], [15].

### Differentiation of Rat iPS Cells *in vitro* and *in vivo*

Rat iPS cell differentiation was investigated *in vitro* by embryoid body (EB) formation, and *in vivo* by teratoma formation in NSG mice.

**Figure 3:**
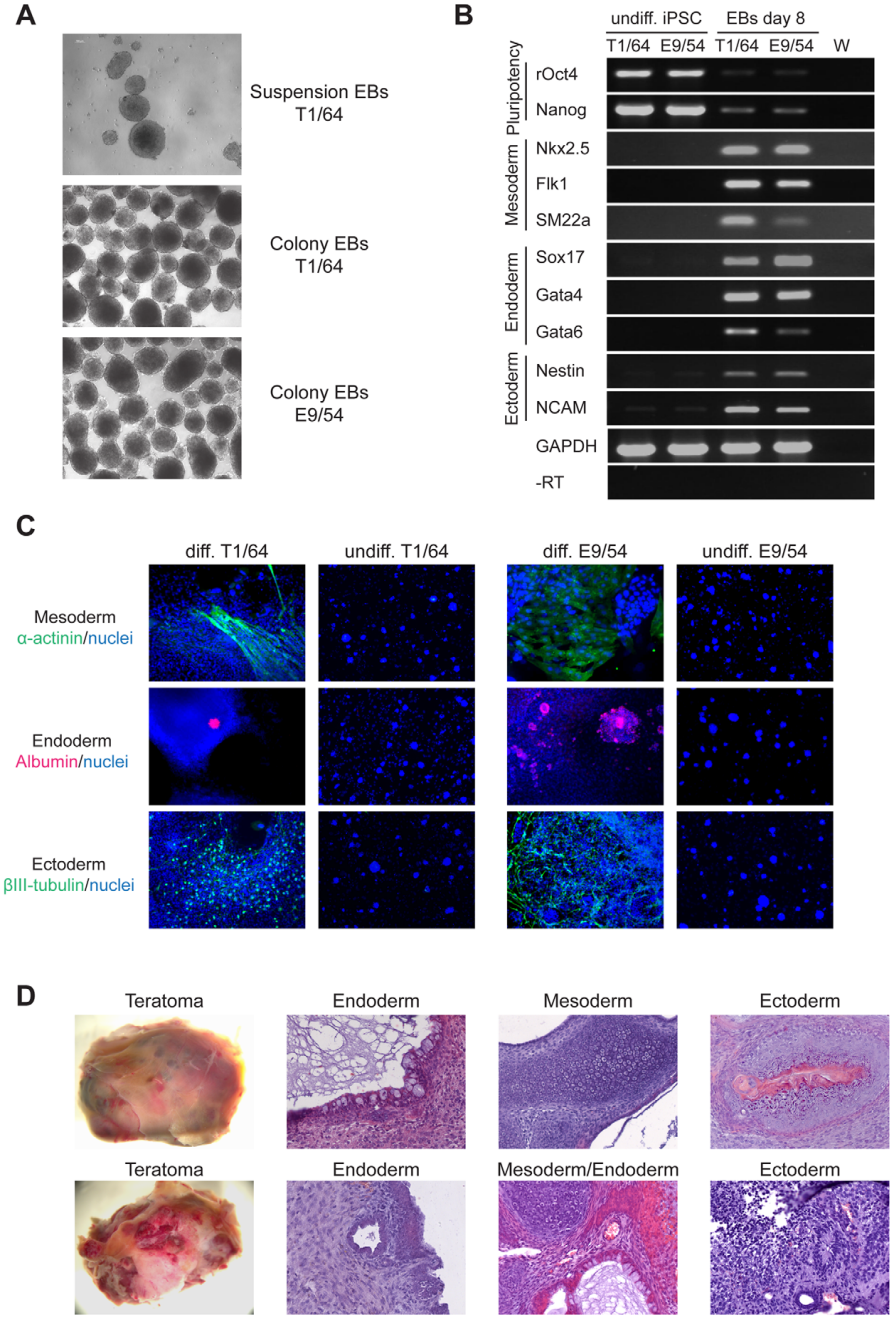
Differentiation analysis of rat iPS cells. (A) Representative images of embryoid bodies (EBs) generated by suspension or colony EB culture of lines T1/64 and E9/54 (10× magnification). (B) RT-PCR analysis of T1/64 and E9/54 before and after EB based differentiation. Marker genes for undifferentiated cells and differentiated derivatives of the three embryonic germ layers are as indicated. GAPDH control, water (W) and reverse transcriptase enzyme (-RT) negative controls are indicated. (C) Immunocytochemical analysis of undifferentiated and differentiated rat iPS cells from line T1/64 and E9/54 (10× magnification). Markers for three germ layers were: albumin, sarcomeric α-actinin, βIII-tubulin. Nuclei were stained with Hoechst. (D) Teratoma analysis. First column: Macroscopic images of one teratoma. Second column: H&E stained section showing endoderm derived intestinal epithelium (top and bottom). Third column: H&E stained section showing mesoderm derivatives cartilage (top and bottom) and blood vessels (bottom); and endoderm derivatives pancreatic tissue and intestinal epithelium (bottom). Fourth column: H&E stained section showing ectoderm derivatives keratinized epidermis (top) and neural rosettes (bottom) (20× magnification).

EB procedures developed for mouse ES cells [34] were unsuccessful when applied unmodified to rat iPS cells. Rat iPS cells cultured as single cells in suspension in medium containing serum typically died after two days. We therefore developed an alternative protocol. Cell suspensions were cultured in 50% DMEM+, 50% N2B27-2i medium (EB medium I) for two days, during which cells spontaneously aggregated. Medium was changed to 70% DMEM+, 30% N2B27-2i (EB medium II) for two days, then to DMEM+. Under these conditions rat iPS cells reliably generated EBs (see Figure 3A). These findings are consistent with reports that the GSK3β inhibitor CHIR99021 is necessary for EB formation in rat ES cells [14]. We also developed a technique, termed colony EB culture, that produced EBs with high efficiency by taking advantage of the poor attachment of undifferentiated rat iPS cells. Colonies 100 to 200 µm in diameter were flushed off the feeder layer, resuspended in EB medium I and cultured further as described above. This improved cell survival (see Figure 3A), but produced EBs of diverse size.

We used colony EB culture with iPS cell line T1/64 and E9/54 and analyzed expression of genes characteristic of the three embryonic germ layers by RT-PCR. After 8 days in suspension, mesodermal (Nkx2.5, Flk1, SM22a), endodermal (Sox17, Gata4, Gata6) and ectodermal (Nestin, NCAM) marker genes were expressed, whereas Oct4 and Nanog expression was markedly reduced (see Figure 3B). EBs were allowed to attach to gelatin-coated plates, cultured for 12 days in medium containing serum (DMEM+) and outgrowths examined. Immunocytochemical analysis revealed differentiation to neurons as detected by expression of βIII-tubulin, cardiomyocytes by sarcomeric α-actinin, and hepatocytes by albumin, see Figure 3C. Undifferentiated rat iPS cells did not express these markers, see Figure 3C.

The ability of rat iPS cells to generate differentiated tissues within a teratoma was tested by injecting undifferentiated T1/64 cells into NSG immunodeficient mice. All injection sites generated tumors up to 1.5 cm in diameter after 25 days. One was sectioned and examined histologically. Staining with H&E revealed complex organized structures and identifiable derivatives of the three embryonic germ layers, including intestinal epithelium and pancreatic cells (endoderm), cartilage and blood vessels (mesoderm), also neural rosettes and epidermis (ectoderm), as shown in Figure 3D. These data demonstrate that our rat iPS cells are pluripotent.

### Rat iPS Cells in Feeder-free Monolayer Culture

**Figure 4:**
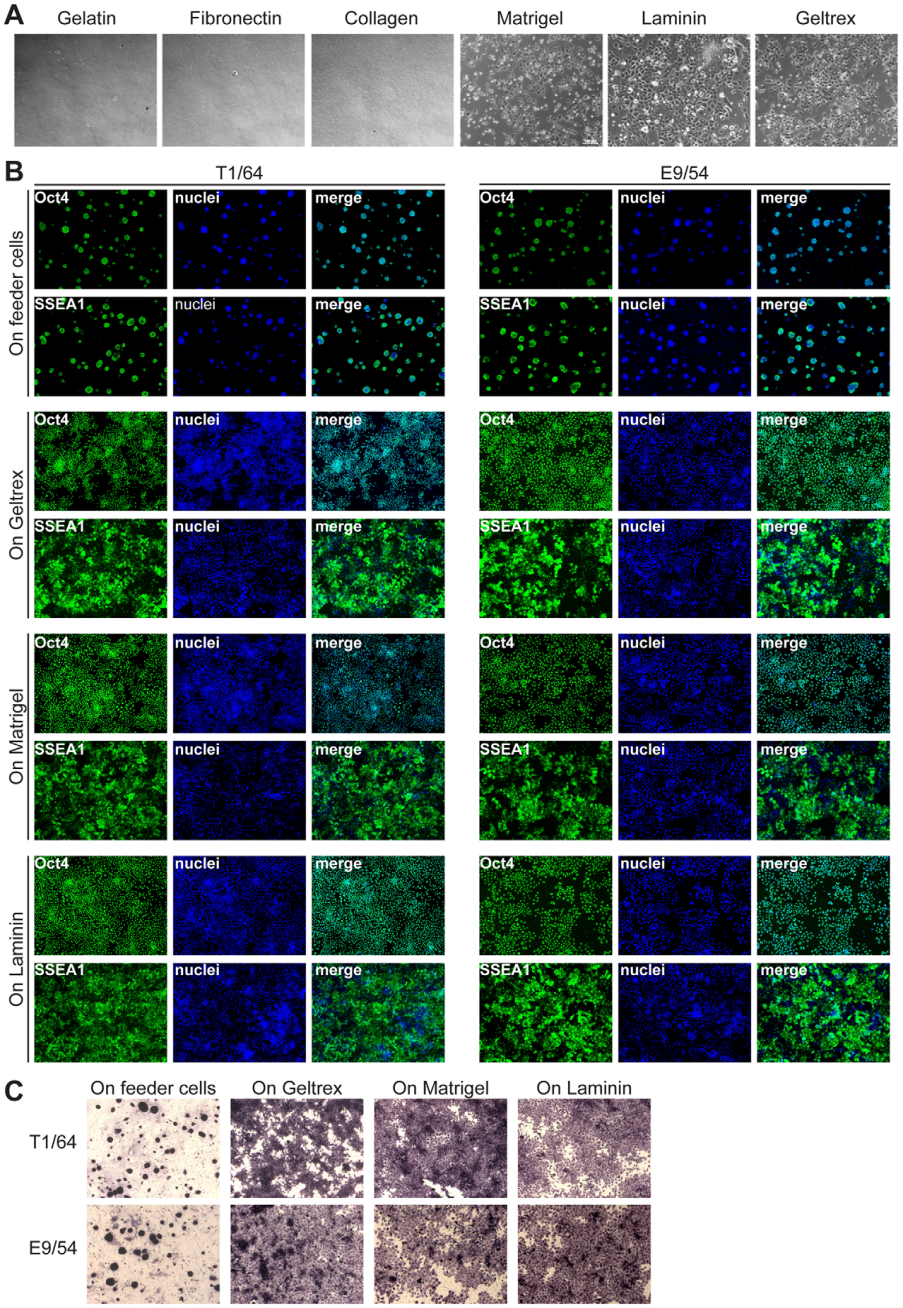
Rat iPS cells in monolayer culture. (A) Attachment of rat iPS cells to coated tissue culture plates (10× magnification). Plates were coated with gelatin, fibronectin, collagen I, Matrigel, laminin or Geltrex. (B) Immunocytochemical analysis of rat iPS cell lines T1/64 and E9/54 cultured on Geltrex, Matrigel, laminin or feeder cells for Oct4, SSEA1 and SSEA4 (10× magnification). (C) Alkaline phosphatase staining of rat iPS cell lines T1/64 and E9/54 cultured on Geltrex, Matrigel, laminin or feeder cells (10× magnification).

We investigated whether our rat iPS cells could be cultured as monolayers without feeder cells. This would allow more control over differentiation than EB based methods. Rat iPS and ES cells are known to attach poorly even on feeder cells, but success has been reported with rat ES cells on laminin coated plates [6]. We compared laminin, bovine gelatin, collagen type I, fibronectin, and the Engelbreth-Holm-Swarm tumor basement membrane extracts Matrigel and Geltrex. No attachment was observed on gelatin, collagen type I or fibronectin. In contrast, rat iPS cells attached to Matrigel, Geltrex or laminin coated plates, proliferated and formed colonies of morphologically undifferentiated cells (see Figure 4A). Cells on Matrigel, Geltrex and laminin were fixed after 5 to 7 days and characterized by immunocytochemistry and alkaline phosphatase (AP) staining. Similar to iPS cells on feeder layers, they were positive for Oct4, SSEA1, AP and negative for SSEA4 (see Figure 4B and 4C). Immunocytochemical detection of differentiation markers albumin, βIII-tubulin and sarcomeric α-actinin was negative, confirming that rat iPS cells on Matrigel, Geltrex and laminin remain undifferentiated (see Figure S1). Cumulative cell numbers and population doubling times were compared between cells grown on Matrigel, Geltrex, laminin and on feeder cells (see Figure S2). No differences were observed after 48 h, but after 96 h iPS cell numbers were higher on Matrigel, Geltrex and laminin (mean 1.75×10^6^ cells per well) than on feeders (1.17×10^6^ cells per well). Doubling times based on exponential cell growth were 16.4 h (laminin), 19.8 h (Matrigel), 20.6 h (Geltrex) and 27.8 h (feeders).

### Transfection and Protein Transduction of Rat iPS Cells

**Figure 5:**
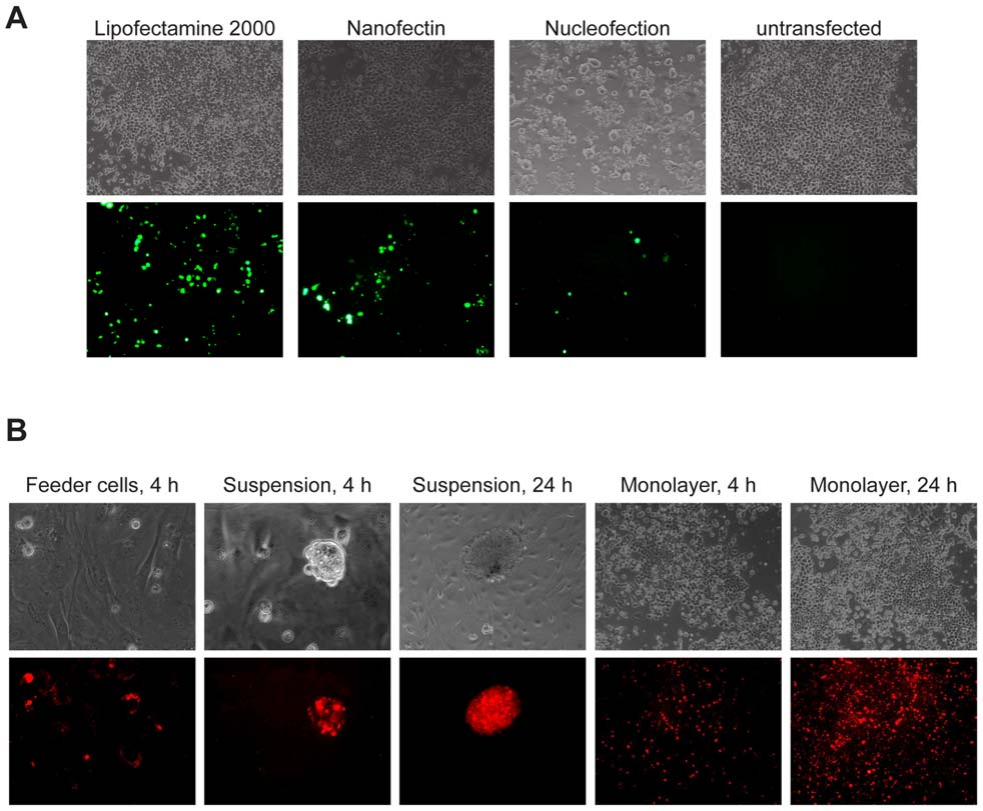
DNA transfection and protein transduction of iPS cells. (A) Rat iPS cells showing GFP expression after transfection with Lipofectamine 2000, Nanofectin (feeder-free monolayer culture) or Nucleofection. Untransfected cells were used as a negative control (10× magnification). (B) Rat iPS cells showing red fluorescence after transduction (4 h or 24 h) with NLS-Cherry-9R protein on feeder cells, in suspension or feeder-free monolayer culture (20× and 10× magnification).

The ability of pluripotent cells to undergo genetic and other manipulations in culture is fundamental to their practical usefulness. We therefore assessed rat iPS cells for their ability to undergo DNA transfection by three different methods: Nanofection and Lipofection of feeder-free monolayers and Nucleofection of suspended cells. A GFP reporter plasmid was used as a convenient indicator. Transient transfection efficiency determined after 24 h showed more than 10% of cells transfected with Lipofectamine 2000, ∼9% with Nanofectin and ∼4% with Nucleofection, see Figure 5A. Cells were replated onto feeders two days after transfection and no change in iPS morphology was observed. Lipofectamine 2000 and Nucleofection slightly reduced cell viability, but Nanofectin had no detectable effect.

We investigated if our rat iPS cells can be efficiently transduced with recombinant proteins. This would for example allow direct delivery of recombinases such as Cre, or transcription factors for directed differentiation without genetic manipulation. A cell penetrating red fluorescent protein, NLS-Cherry-9R, was generated by fusing the mCherry coding region to a protein transduction domain consisting of 9 arginine residues (9R) and the SV40 Large-T nuclear localization signal (NLS) [35], [36], [37], [38]. NLS-Cherry-9R was expressed in bacteria, and rat iPS cells transduced for either 4 or 24 hours on feeder cells, in suspension or as feeder-free monolayers. As shown in Figure 5B, 24 hours transduction in suspension or monolayer culture resulted in up to 80% red fluorescent cells.

These experiments demonstrate that our rat iPS cells are amenable to genetic and non-genetic manipulation, enabling the use of a wide range of molecular tools.

## Discussion

We describe a significant advance in rat iPS technology, the efficient generation of rat iPS cells using a single non-viral vector that allows tight control over reprogramming factor expression. The established iPS lines were self-sustaining and had activated the endogenous pluripotency program. Methods have been developed to improve rat iPS cell viability and successful generation of differentiated iPS derivatives *in vitro*. Teratoma formation *in vivo* and differentiation *in vitro* demonstrate pluripotency. Our rat iPS cells can be cultured as monolayers free of feeder cells, at least for short periods, and readily undergo DNA transfection and protein transduction.

To date rat iPS cells have been generated using retroviral or lentiviral vectors based on the original breakthrough by Yamanaka [7], [8], [15], [16], [9]. However, the viral approach has shortcomings. To circumvent the need for multiple viral infections, Hamanaka *et al.* (2011) developed a polycistronic inducible lentiviral system that encodes all factors [39]. This enables control of factor expression and introduces individual factors in equivalent ratio. Nevertheless lentiviral transduction still requires high viral titers for efficient transduction and viral production must be under strict biosafety conditions.

Our plasmid based reprogramming approach avoids these issues. A single vector contains the reprogramming factors under doxycycline-inducible control and all necessary Tet-On regulatory components. Because transfected DNA integrates into the host genome less frequently than infecting retro- or lentiviruses [40], an attB site was included to facilitate φC31 integrase-mediated integration [41], [24]. Cotransfection of the reprogramming vector with φC31 integrase enabled efficient generation of doxycycline-dependent rat iPS cells. Direct comparison of the efficiency of our approach with that of others is not always straightforward because many reports base their calculations on different parameters, such as the number of stable iPS cell lines, alkaline phosphatase positive colonies, SSEA1 positive colonies or cell morphology (see Table 3). Reprogramming efficiency, calculated as the number of stable iPS cell lines obtained relative to starting cell number, reveals a range of 0.00174% to 0.014% in previous reports [7], [15], [42], [43]. We obtained 0.0027% to 0.0028% for doxycycline-dependent and 0.005% to 0.0078% for doxycycline-independent rat iPS cells. This compares favorably with methods based on viral vectors.

Generation of self-sustaining rat iPS cells after doxycycline removal depended on the culture medium. N2B27-2i medium was superior to N2B27-3i at this stage, highlighting a negative influence of the ALK5 inhibitor A83-01 or Knockout Serum Replacement on iPS generation. This indicates that one or both reduces colony formation, maintenance and expansion and accords with previous reports that N2B27-2i conditions are suitable for rat ES and iPS culture [5], [6], [15]. Addition of Thiazovivin, ROCK inhibitor Y27632 or ascorbic acid did not increase efficiency compared to N2B27-3i or N2B27-2i.

Traditional methods of EB formation such as hanging drops, or aggregation in single cell suspension are difficult with rat pluripotent stem cells [5], [14], [15]. Our colony EB method is a significant improvement that reduces cell death and allows reliable generation of EBs without the need for additional small molecules such as the ROCK inhibitor Y27632 [14].

We also investigated appropriate conditions for feeder-free monolayer culture and found that rat iPS cells attached to Matrigel, Geltrex and laminin substrates. Previously only attachment to laminin has been shown [6]. Rat iPS cells grown on Matrigel, Geltrex or laminin maintained expression of Oct4, SSEA1 and alkaline phosphatase. Cumulative cell numbers were similar on the three substrates but slight differences were observed in population doubling time. The increased range of coating matrices opens new possibilities for feeder-free iPS cell culture, transfection and cell differentiation without EB formation.

Future applications such as gene targeting require genetic manipulation, preferably free of feeder cells. The established monolayer conditions enabled transfection with Lipofectamine 2000 and Nanofectin, resulting in up to 10% transfection efficiency, useful alternatives to the most commonly used techniques, nucleofection and electroporation [5], [44], [45], [11].

Directed differentiation of iPS cells may require the addition of inductive transcription factors for only a short time. Transduction of protein rather than DNA is a useful means of achieving this, and also leaves the genome unaltered. Our rat iPS cells can be efficiently transduced with recombinant proteins in suspension or monolayer culture. Rat iPS cells can thus be cultured on feeder cells, transfected/transduced in monolayer culture and then transferred back onto feeder cells as required.

We have demonstrated that rat iPS cells can be generated by a safe and simple non-viral approach. Improved methods for differentiation and the use of coating substrates for monolayer culture will facilitate derivation of many different cell types from rat iPS and ES cells, a goal not yet achieved. These will provide valuable resources for diverse biomedical research.

## Supporting Information

Figure S1 Immunocytochemical analysis of undifferentiated rat iPS cells. Immunocytochemical analysis of rat iPS cell lines T1/64 and E9/54 cultured on Geltrex, Matrigel, laminin or feeder cells for albumin, sarcomeric α-actinin (Actinin) and βIII-tubulin (TuJ1) (10× magnification).
(TIF)

Figure S2 Cumulative cell number of rat iPS cells on different matrices. Cell numbers on day 0, 2 and 4 of rat iPS cells cultured on Geltrex, Matrigel, laminin or feeder cells. Total cell number per well of a 12 well plate was determined in triplicate.
(TIF)

Table S1 Oligonucleotides for RT-PCR and qRT-PCR.
(DOC)

Table S2 Oligonucleotides for integration analysis.
(DOC)

Table S3 Oligonucleotides for bisulfite sequencing and Southern blot analysis.
(DOC)

Table S4 Different culture media for rat iPS cells.
(DOC)
