# Supplementary figures and images for: Efficient Generation of Rat Induced Pluripotent Stem Cells Using a Non-Viral Inducible Vector
Source: PLoS One. 2013 Jan 31;8(1):e55170. doi: 10.1371/journal.pone.0055170 (PMC3561372; doi:10.1371/journal.pone.0055170)

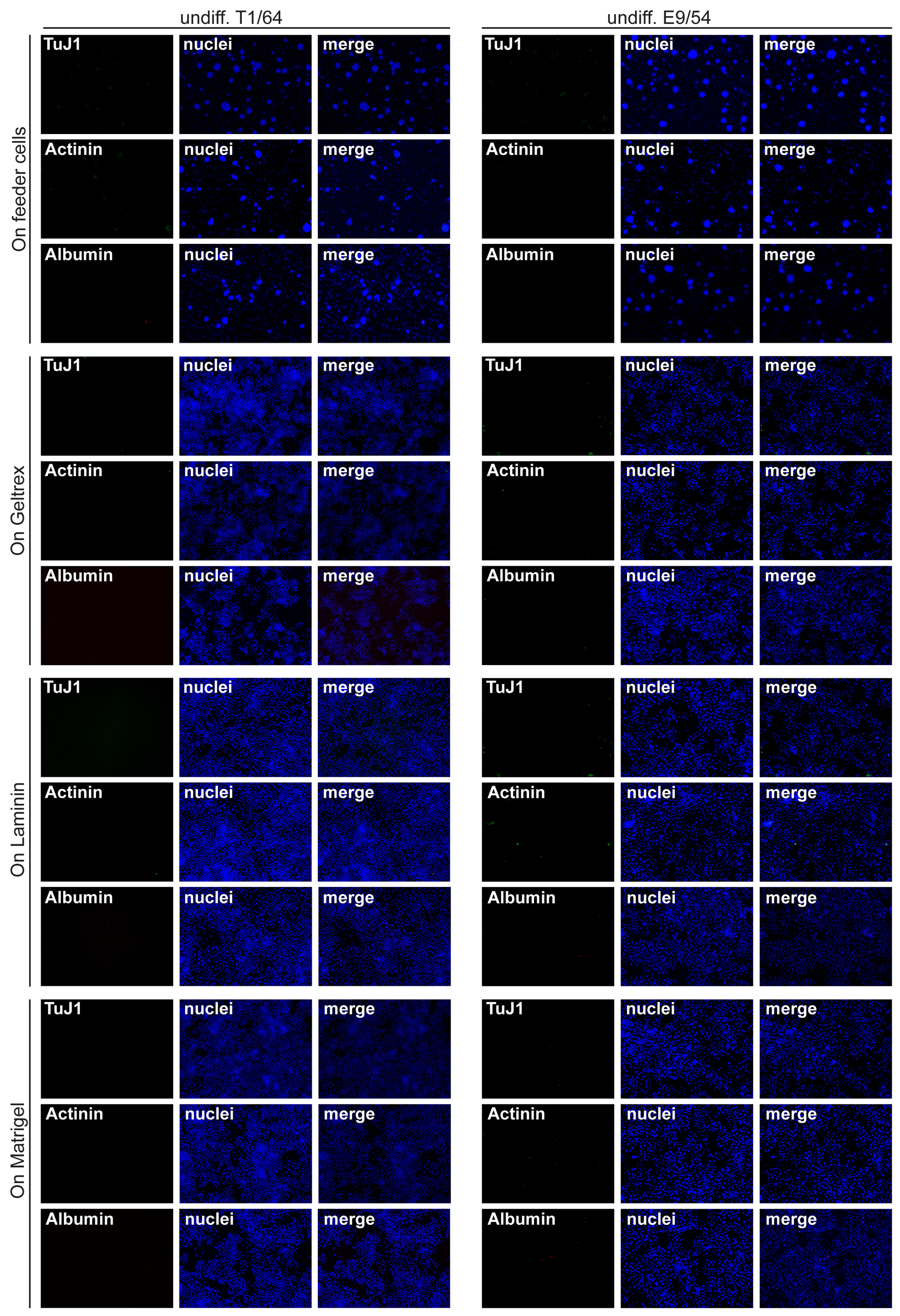

Supplement: Figure S1 — Immunocytochemical analysis of undifferentiated rat iPS cells. Immunocytochemical analysis of rat iPS cell lines T1/64 and E9/54 cultured on Geltrex, Matrigel, laminin or feeder cells for albumin, sarcomeric α-actinin (Actinin) and βIII-tubulin (TuJ1) (10× magnification). (TIF) [file pone.0055170.s001.tif]

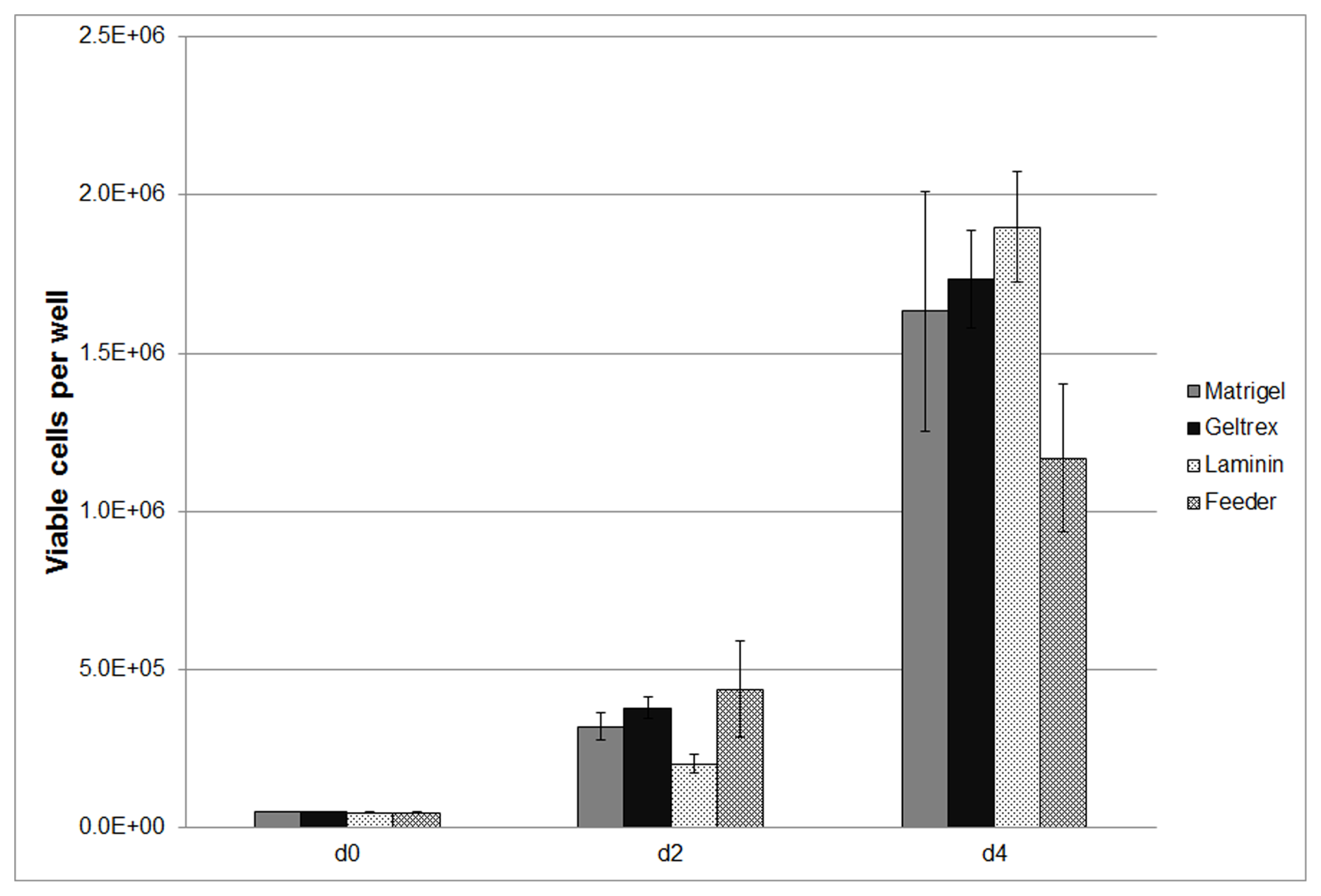

Supplement: Figure S2 — Cumulative cell number of rat iPS cells on different matrices. Cell numbers on day 0, 2 and 4 of rat iPS cells cultured on Geltrex, Matrigel, laminin or feeder cells. Total cell number per well of a 12 well plate was determined in triplicate. (TIF) [file pone.0055170.s002.tif]
