# Supplementary material for: Efficient Generation of Rat Induced Pluripotent Stem Cells Using a Non-Viral Inducible Vector
Source: PLoS One. 2013 Jan 31;8(1):e55170. doi: 10.1371/journal.pone.0055170 (PMC3561372; doi:10.1371/journal.pone.0055170)
Supplement: Table S1 — Oligonucleotides for RT-PCR and qRT-PCR. (DOC) [file pone.0055170.s003.doc]

Supplementary Table S1: Oligonucleotides for RT-PCR and qRT-PCR

| Name | Sequence (5’-3’) | Product size |
| --- | --- | --- |
| **RT-PCR** | | |
| rOct4_F | AGAACCGTGTGAGGTGGAAC | 423 bp |
| rOct4_R | TGTCTACCTCCCTTCCTTGC |  |
| Nanog_F | GTACCTCAGCCTCCAGCAGA | 503 bp |
| Nanog_R | GGCTTTCCCTAGTGGCTTCC |  |
| rSox2_F | cacaactcggagatcagcaa | 820 bp |
| rSox2_R | ccctcccaattcccttgtat |  |
| Rex1_F | TTCTTGCCAGGTTCTGGAAGC | 301 bp |
| Rex1_R | TTTCCCACACTCTGCACACAC |  |
| FGF4_F | tgtggtgagcatcttcggagtgg | 197 bp |
| FGF4_R | ccttcttggtccgcccgttctta |  |
| GAPDH_F | CTTCATTGACCTCAACTAC | 593/881* bp |
| GAPDH_R | GGAAGGCCATGCCAGTGAGC |  |
| Nkx2.5_F | CGGTGGAGCTGGACAAAGCC | 217 bp |
| Nkx2.5_R | TAGCGGCGGTTCTGGAACCA |  |
| Flk1_F | ATACACCTGCACAGCGTACAG | 271 bp |
| Flk1_R | TCCCGCATCTCTTTCACTCAC |  |
| SM22a_F | GCTGAAGAATGGCGTGATTCTGAG | 195 bp |
| SM22a_R | CCTTCAAAGAGGTCAACAGTCTGG |  |
| Sox17_F | AGGAGAGGTGGTGGCGAGTAG | 268 bp |
| Sox17_R | GTTGGGATGGTCCTGCATGTG |  |
| Gata4_F | CGCTTACACCCCACCGCCTG | 478 bp |
| Gata4_R | AGGCTTGATGAGGGGCCGGT |  |
| Gata6_F | TCATCACGACGGCTTGGACTG | 467 bp |
| Gata6_R | GCCAGAGCACACCAAGAATCC |  |
| Nestin_F | AGAGAAGCGCTGGAACAGAG | 234 bp |
| Nestin_R | AGGTGTCTGCAACCGAGAGT |  |
| NCAM_F | TGCTCAAGTCCCTAGACTGGAACG | 452 bp |
| NCAM_R | CTTCTCGGGCTCTGTCAGTGGTGTGG |  |
| **qRT-PCR** | | |
| qGAPDH_F | GACATGCCGCCTGGAGAAAC | 92 bp |
| qGAPDH_R | AGCCCAGGATGCCCTTTAGT |  |
| qOct4/c-myc_F | CCACTTCACCACACTCTACT | 127 bp |
| qOct4/c-myc_R | GTCACCGCATGTTAGAAGAC |  |
| qSox2/Klf4_F | CGCATGTTAGAAGACTTCCT | 166 bp |
| qSox2/Klf4_R | GACATGATCAGCATGTACCT |  |

*: fragment size from genomic DNA
