# Supplementary material for: Efficient Generation of Rat Induced Pluripotent Stem Cells Using a Non-Viral Inducible Vector
Source: PLoS One. 2013 Jan 31;8(1):e55170. doi: 10.1371/journal.pone.0055170 (PMC3561372; doi:10.1371/journal.pone.0055170)
Supplement: Table S2 — Oligonucleotides for integration analysis. (DOC) [file pone.0055170.s004.doc]

Supplementary Table S2: Oligonucleotides for integration analysis

| Name | Sequence (5’-3’) |
| --- | --- |
| rps1F | tccttacccgtcatcgc |
| rps1R | tacggagatggtggttgcactg |
| rps2F | gcccctcccatctatctcta |
| rps2R | acgtgcccatgacacacctt |
| rps3_F | gccccagagcaacacctt |
| rps3R | catacccacagtggtcagacgg |
| ReB | GGACCACCTTGCCTTACACA |
