# Supplementary material for: Efficient Generation of Rat Induced Pluripotent Stem Cells Using a Non-Viral Inducible Vector
Source: PLoS One. 2013 Jan 31;8(1):e55170. doi: 10.1371/journal.pone.0055170 (PMC3561372; doi:10.1371/journal.pone.0055170)
Supplement: Table S3 — Oligonucleotides for bisulfite sequencing and Southern blot analysis. (DOC) [file pone.0055170.s005.doc]

Supplementary Table S3: Oligonucleotides for bisulfite sequencing and Southern blot analysis

| Primer name | Sequence (5’-3’) | Product size |
| --- | --- | --- |
| BS-Oct4_F | atgggattttggaggatttttag | 206 bp |
| BS-Oct4_R | ctcaaacccaaatacccctactt |  |
| prKlf4_F | agtgcctggtcagttcatcc | 1308 bp |
| prKlf4_R | ctctgctcccgtccttctc |  |
