# Supplementary material for: Efficient Generation of Rat Induced Pluripotent Stem Cells Using a Non-Viral Inducible Vector
Source: PLoS One. 2013 Jan 31;8(1):e55170. doi: 10.1371/journal.pone.0055170 (PMC3561372; doi:10.1371/journal.pone.0055170)
Supplement: Table S4 — Different culture media for rat iPS cells. (DOC) [file pone.0055170.s006.doc]

Supplementary Table S4: Different culture media for rat iPS cells

| Name | Content | Description |
| --- | --- | --- |
| Medium 1 | N2 medium, B27 medium (1:1)  3 µM GSK3β inhibitor CHIR99021  0.5 µM MEK1/2 inhibitor PD0325901  0.5 µM ALK5 inhibitor A83-01  20% Knockout serum replacement  1000 U/ml hLIF  0.1 mM beta-mercaptoethanol | N2B27-3i medium |
| Medium 2 | N2 medium, B27 medium (1:1)  3 µM GSK3β inhibitor CHIR99021  0.5 µM MEK1/2 inhibitor PD0325901  0.5 µM ALK5 inhibitor A83-01  1000 U/ml hLIF  0.1 mM beta-mercaptoethanol | N2B27-3i medium without Knockout serum replacement |
| Medium 3 | N2 medium, B27 medium (1:1)  3 µM GSK3β inhibitor CHIR99021  0.5 µM MEK1/2 inhibitor PD0325901  20% Knockout serum replacement  1000 U/ml hLIF  0.1 mM beta-mercaptoethanol | N2B27-2i medium with Knockout serum replacement |
| Medium 4 | N2 medium, B27 medium (1:1)  3 µM GSK3β inhibitor CHIR99021  0.5 µM MEK1/2 inhibitor PD0325901  1000 U/ml hLIF  0.1 mM beta-mercaptoethanol | N2B27-2i medium |
| Medium 5 | N2 medium, B27 medium (1:1)  1 µM Thiazovivin  0.5 µM MEK1/2 inhibitor PD0325901  20% Knockout serum replacement  1000 U/ml hLIF  0.1 mM beta-mercaptoethanol | Replacement of GSK3β inhibitor CHIR99021 with Thiazovivin, with Knockout serum replacement |
| Medium 6 | N2 medium, B27 medium (1:1)  1 µM Thiazovivin  0.5 µM MEK1/2 inhibitor PD0325901  1000 U/ml hLIF  0.1 mM beta-mercaptoethanol | Replacement of GSK3β inhibitor CHIR99021 with Thiazovivin, without Knockout serum replacement |
| Medium 7 | N2 medium, B27 medium (1:1)  3 µM GSK3β inhibitor CHIR99021  0.5 µM MEK1/2 inhibitor PD0325901  0.5 µM ALK5 inhibitor A83-01  20% Knockout serum replacement  1000 U/ml hLIF  0.1 mM beta-mercaptoethanol  50 µg/ml ascorbic acid | N2B27-3i medium with ascorbic acid |
| Medium 8 | N2 medium, B27 medium (1:1)  3 µM GSK3β inhibitor CHIR99021  0.5 µM MEK1/2 inhibitor PD0325901  0.5 µM ALK5 inhibitor A83-01  1000 U/ml hLIF  0.1 mM beta-mercaptoethanol  50 µg/ml ascorbic acid | N2B27-3i medium without Knockout serum replacement, with ascorbic acid |
| Medium 9 | N2 medium, B27 medium (1:1)  3 µM GSK3β inhibitor CHIR99021  0.5 µM MEK1/2 inhibitor PD0325901  0.5 µM ALK5 inhibitor A83-01,  10 µM ROCK inhibitor Y-27632  20% Knockout serum replacement  1000 U/ml hLIF  0.1 mM beta-mercaptoethanol | N2B27-3i medium with ROCK inhibitor Y-27632 |
| Medium 10 | N2 medium, B27 medium (1:1)  3 µM GSK3β inhibitor CHIR99021  0.5 µM MEK1/2 inhibitor PD0325901  0.5 µM ALK5 inhibitor A83-01,  10 µM ROCK inhibitor Y-27632  1000 U/ml hLIF  0.1 mM beta-mercaptoethanol | N2B27-3i medium without Knockout serum replacement, with ROCK inhibitor Y-27632 |
| Medium 11 | N2 medium, B27 medium (1:1)  3 µM GSK3β inhibitor CHIR99021  0.5 µM MEK1/2 inhibitor PD0325901  20% Knockout serum replacement  0.1 mM beta-mercaptoethanol | N2B27-2i medium with Knockout serum replacement, without hLIF |
| Medium 12 | N2 medium, B27 medium (1:1)  3 µM GSK3β inhibitor CHIR99021  0.5 µM MEK1/2 inhibitor PD0325901  0.1 mM beta-mercaptoethanol | N2B27-2i medium without hLIF |
